# Electromyographic Patterns and the Identification of Subtypes of Awake Bruxism

**DOI:** 10.3389/fnhum.2020.601881

**Published:** 2021-01-28

**Authors:** Ubirakitan Maciel Monteiro, Vinicius Belém Rodrigues Barros Soares, Caio Belém Rodrigues Barros Soares, Tiago Coimbra Costa Pinto, Rosana Christine Cavalcanti Ximenes, Marcelo Araújo Cairrão Rodrigues

**Affiliations:** Federal University of Pernambuco, Recife, Brazil

**Keywords:** awake bruxism, electromyography, temporal muscle, cluster analysis, tonic contraction, phasic contractions

## Abstract

The future of awake bruxism assessment will incorporate physiological data, possibly electromyography (EMG) of the temporal muscles. But up to now, temporal muscle contraction patterns in awake bruxism have not been characterized to demonstrate clinical utility. The present study aimed to perform surface EMG evaluations of people assessed for awake bruxism to identify possible different subtypes. A 2-year active search for people with awake bruxism in three regions of the country resulted in a total of 303 participants (223 women, 38 ± 13 years, mean and SD). Their inclusion was confirmed through non-instrumental approaches for awake bruxism: self-reported questionnaire and clinical exam, performed by three experienced and calibrated dentists (Kappa = 0.75). Also, 77 age- and sex-matched healthy controls were recruited (49 women, 36 ± 14 years). Temporalis surface EMG was performed with a portable device (Myobox; NeuroUp, Brazil). EMG signals were sent to a computer via Bluetooth 4.0 at a sampling rate of 1,000 Hz. Digital signal processing was performed using the commercial neuroUP software, transformed in RMS and then normalized for peak detection (EMG peaks/min), in a 10 min session. Cluster analysis revealed three distinct subtypes of awake bruxism: phasic, tonic, and intermediate. Individuals with a predominance of EMG peaks/min were classified as the “phasic” subtype (16.8%). Those with the highest EMG rest power were classified as the “tonic” subtype (32.3%). There was also an “intermediate” subtype (50.8%), when both variables remained low. Characterization of awake bruxism physiology is important for future establishment of instrumental assessment protocols and treatment strategies.

## Introduction

Bruxism is an umbrella term for different motor phenomena/behaviors of the masticatory muscles. An international consensus defined awake bruxism as a masticatory muscle activity behavior during wakefulness that is characterized by repetitive or sustained tooth contact and/or by bracing or thrusting of the mandible (Lobbezoo et al., 2018). Awake bruxism is not defined as a disease by the current consensus (Lobbezoo et al., 2018) and does not necessarily present with pain at the time it is identified. But it is important to study this phenomenon because it is known that people with awake bruxism have a greater probability of suffering from temporomandibular pain and diseases in the future (Maltarollo et al., 2020; Wetselaar et al., 2020).

The prevalence of awake bruxism in the general population is high (8–31%), and is influenced by psychological factors, such as stress and anxiety, a quite common situation in the post COVID-19 pandemy (Manfredini et al., 2013a, 2015; Lobbezoo et al., 2018; Winocur et al., 2019). The new consensus in bruxism (Lobbezoo et al., 2018) states that assessment of this condition in the future will need to go beyond clinical evaluation. Physiological parameters are needed, and electromyography (EMG) was identified as an alternative, despite the fact that no protocols have been suggested yet. Also, a recent review of literature (Yamaguchi et al., 2020) stated that masticatory muscle EMG can be easily and precisely recorded during the daytime by using a wearable EMG device that improves the assessment of awake bruxism. However, there are no studies regarding the physiology of awake bruxism using EMG. This knowledge is necessary to advance the field and for future long-term follow-up studies of awake bruxism based on physiological data.

Surface EMG is a widely used non-invasive technique capable of amplifying electrical signals captured on the skin above superficial muscles. The signals, which are conducted through the tissues and captured by electrodes, represent the temporal and spatial summation of a population of nearby motor units (Rainoldi et al., 2004). Therefore, the present study aimed to perform EMG evaluations of people included in the assessment of awake bruxism. Our hypothesis is that there are different patterns of activation of the masticatory muscles in awake bruxism and that these profiles can be unveiled by EMG assessment. We also hypothesized that pain intensity levels would be different among the EMG-based profiles for awake bruxism, such that participants with tonic temporal muscle contractions would report the highest pain intensity levels.

## Methods

An observational, analytic, and cross-sectional study was conducted. The participants were recruited by convenience from private healthcare services over 2 years (2017–2019) in the northeastern, central-western, and southeastern regions of Brazil. An active search was conducted in all centers to recruit subjects with a high probability of awake bruxism. The clinical signs of awake bruxism were disclosed. Possible candidates to the research study came spontaneously or were recruited by clinicians for an evaluation by a specialist. Only those volunteers compatible with the international consensus (Lobbezoo et al., 2018) were included in the awake bruxism group (*n* = 303, 38 ± 14 years, mean, and standard deviation, both sexes). A control group consisted of 77 healthy, age-matched volunteers (36 ± 14 years, both sexes) who were evaluated by the same specialists. All volunteers signed a statement of informed consent in compliance with Resolution n° 466/12 of the National Board of Health, and this study received approval from the human research ethics committee of the Center of Health Sciences of the Federal University of Pernambuco (process 65515417.1.0000.5208).

The inclusion criteria for the awake bruxism group was clinical and followed the international consensus (1): presence of self-reported jaw movements with or without associated pain symptoms, and (2) orofacial clinical signs identified by a dentist. The study exclusion criteria were: history of head trauma, presence of degenerative neurological diseases, stroke, open lesions in the region of the anterior temporal muscle, severe visual or hearing impairment, previous botulinum toxin injection in the masticatory muscles (masseter and temporal), or having undergone other concomitant rehabilitation treatments in the previous month.

All subjects completed a self-reported questionnaire on oral history and masticatory muscle activity, jaw/teeth clenching and bracing/thrusting, and teeth touching during non-swallowing behaviors. Also, a clinical exam was performed by 3 dentists with at least 5 years of experience in clinical assessment of awake bruxism to visually identify masticatory muscle hypertrophy, indentations on the tongue or lip and/or a *linea alba* on the inner cheek, damage to the dental hard tissues, failures of restorative work/prosthodontic constructions, or mechanical wear of the teeth. The Tooth Wear Evaluation System (TWES), recommended by Wetselaar and Lobbezoo (2016), was used as reference. Despite all three specialists being experienced, a calibration procedure was executed as a guarantee that all evaluations would be compatible. The consensus (Lobbezoo et al., 2018), examination criteria, sequence of the examination, and the written specifications were reviewed again. Then, a pre-calibration session included a visual data bank with 30 images of oral alterations of awake bruxism or healthy controls obtained from previous work of the group (Ximenes et al., 2010). After that, a clinical practice calibration session was performed in patients assessed for awake bruxism and controls (*n* = 30 in each group). The three dentists examined the same patients. The data were analyzed, reviewed, and discussed; discrepancies in the data were re-examined. Finally, a reliability session was conducted, and the kappa index was calculated. The calibration procedure continued until a kappa index of 0.75 was reached, indicating an excellent agreement among the three dentists (Landis and Koch, 1997).

Surface EMG was performed using the Myobox device (neuroUP, Brazil). EMG signals were sent to a computer via Bluetooth 4.0 at a sampling rate of 1,000 Hz. Digital signal processing was performed using commercial software (neuroUP, Brazil), with a 60-Hz Notch filter to avoid electrical artifacts and a Butterworth bandpass filter (30–500 Hz) to reduce low frequencies artifacts (e.g., cardiac activity) and to guarantee a reading of up to half of the sampling rate (Nyquist Theorem). The signals were transformed in real-time to calculate the root mean square (RMS). Before the evaluation, an automatic measurement of the signal quality was performed in the software. EMG data were also processed by an algorithm that automatically calculates the amount of electric field gradients per minute. The counting of peaks per minute was based on individualized normalization, not on absolute values. Sudden increases in EMG signal above 80% of the patient's own basal was identified as a peak. This cutoff value was defined in a pilot project carried out prior to the research and was the most accurate value in the identification of phasic contractions.

**Figure 1:**
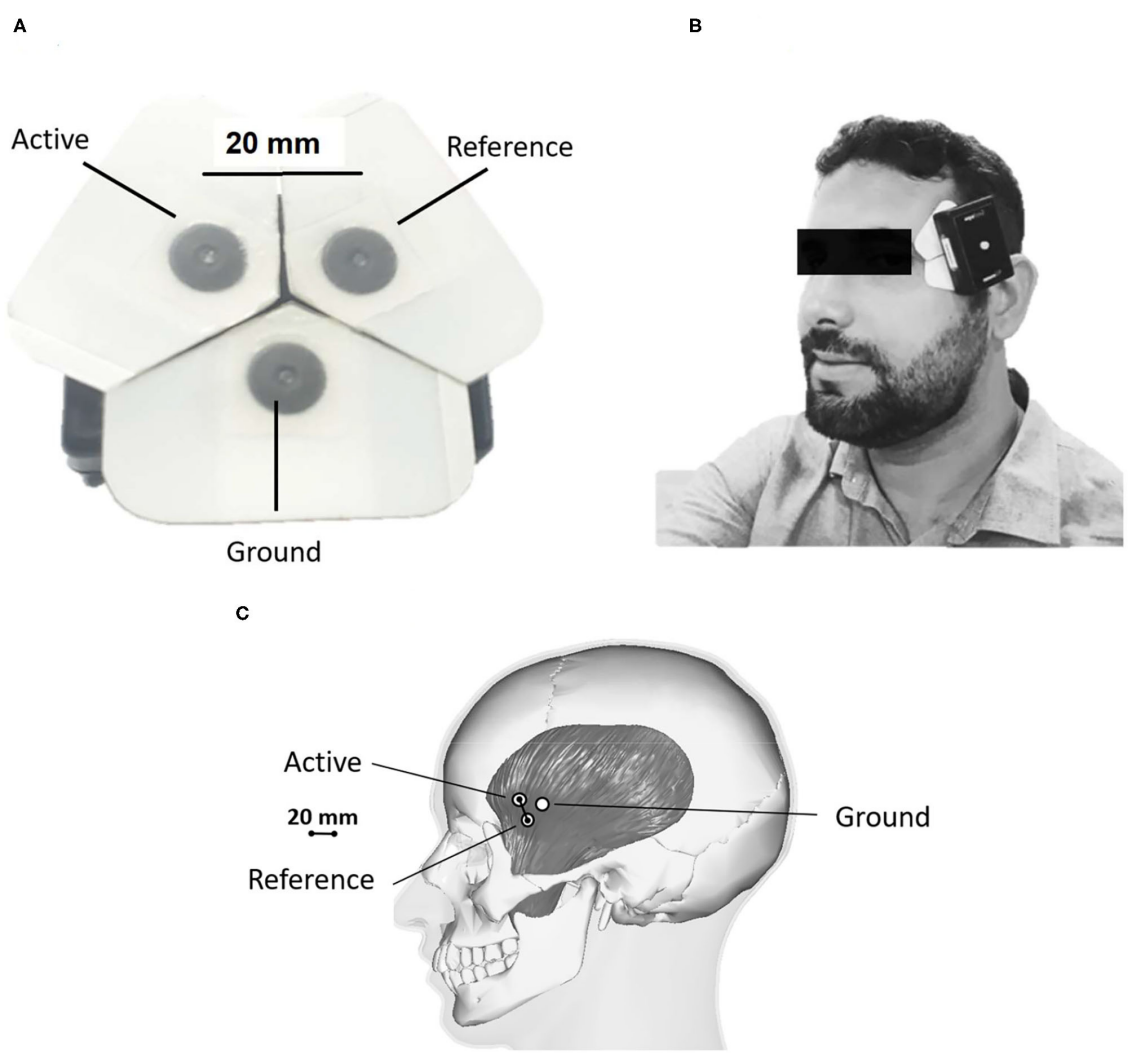
Electrode placement. **(A)** Arrangement of adhesive EMG electrodes; **(B,C)** Positioning of the EMG equipment over the anterior portion of the temporal muscle; The difference between electrodes (20 mm) allows them to be used in volunteers irrespective of age and sex. The white paper surrounding the electrodes can be cut if necessary to fit different head sizes.

Standard disposable surface adhesive electrodes with Ag/AgCl sintered composition were used. These electrodes were placed with a distance of 20 mm between them (Figure 1A). In addition, a ground electrode was positioned 20 mm away from each active electrode (triangulation between them). The sensor was positioned on the anterior portion of the temporal muscle on the right or left side (Figures 1B,C). The side was chosen based on the following criteria: pain upon palpation (first criteria); self-reported habitual chewing side (second criteria). This approach was for operational purposes, as there is no description that awake bruxism has a unilateral effect. Also, a study has shown that people with unilateral masticatory muscle pain show no significant difference in muscle electrical activity of painful and non-painful muscles (Manfredini et al., 2013b).

The EMG procedure lasted ~10 min. Pilot studies from our group (data not shown) and also studies from other laboratories have shown that this period suffices for an EMG evaluation (Prasad et al., 2019). The participant was seated in a comfortable armchair, legs uncrossed, shoulders relaxed, teeth not in contact, back erect, shoulders in external rotation, forearms in supination, and hands resting on the thighs. During the instrumental approach, the EMG signals were observed only by the examiners; the participants did not receive EMG biofeedback.

The Visual Analog Scale (VAS) for pain was used to measure pain intensity. The VAS used was unidimensional, and the volunteer marked the intensity of pain, from zero (no pain) to ten (intense pain). This scale was applied before palpation to avoid trigger points being activated, and participants were instructed to respond about the current painful perception felt at the time of the assessment.

As the objective of present work was to search for profiles of EMG markers in volunteers with awake bruxism, it was decided to perform a cluster analysis. Cluster analysis is a statistical method for organizing data into relatively homogeneous groups that differ from each other according to the variables of interest. All data were plotted in a 2D chart with phase contractions (EMG peaks/min, y) against their rest EMG power (uV RMS). These data were inserted in the automatic cluster analysis of SPSS software, using hierarchical cluster analysis (Crum et al., 2020). The level of significance was set to 5% (*p* < 0.05), and 95% confidence intervals were calculated.

## Results

**Figure 2:**
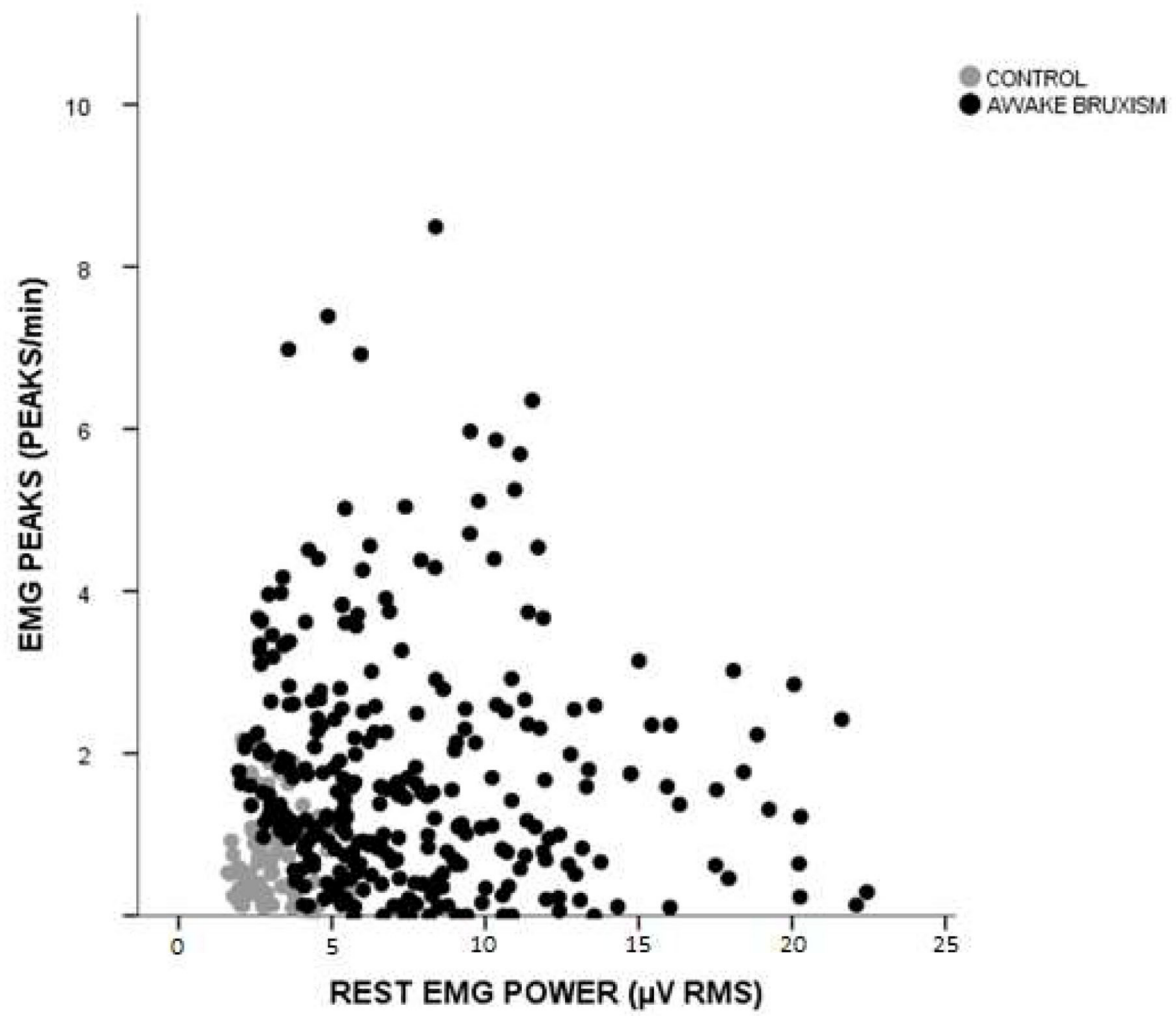
Distribution of participants according to the number of phasic contractions and EMG power at rest. Gray circles: control group (individuals without awake bruxism; *n* = 77). Black circles: awake bruxism group (*n* = 308). EMG, electromyography; RMS, Root mean square.

The age of the 380 participants with and without a clinical evidence of bruxism ranged from 11 to 75 years and included both sexes. The 303 participants with awake bruxism, with or without pain, were considered the participant group. The remaining 77 participants were considered the control group. There were no statistically significant differences among the control and participant groups relative to gender and age (Table 1). Figure 2 shows the EMG peaks per minute and rest muscle power from participant and control groups.

**Table 1:** Descriptive statistics of the study participants.

**Group**	**Age (mean ± SD)**	**Min/Max (years)**	**95% CI lower/upper**	**VAS (arbitrary units 0–10)**	**Rest EMG Power (μV RMS)**	**EMG peaks (peaks/min)**	***n* (male/female)**
Phasic	36 ± 14	6/65	32/40	3.5 ± 3.2	6.4 ± 2.9	4.4 ± 1.3	51 (14/37)
Tonic	39 ± 14	12/75	36/42	3.9 ± 3.1	13.1 ± 4.1	1.1 ± 0.9	98 (22/76)
Intermediate	38 ± 13	11/70	33/39	4.1 ± 3.1	5.1 ± 1.6	1.2 ± 0.7	154 (44/110)
Controls	36 ± 14	13/75	36/39	0.5 ± 0.4	2.9 ± 0.1	0.7 ± 0.5	77 (28/49)

*Values represent mean and standard deviation; 95% CI: confidence interval, interval where 95% of data is located; VAS, visual analog pain scale (0 means no pain); n, group sample size*.

**Figure 3:**
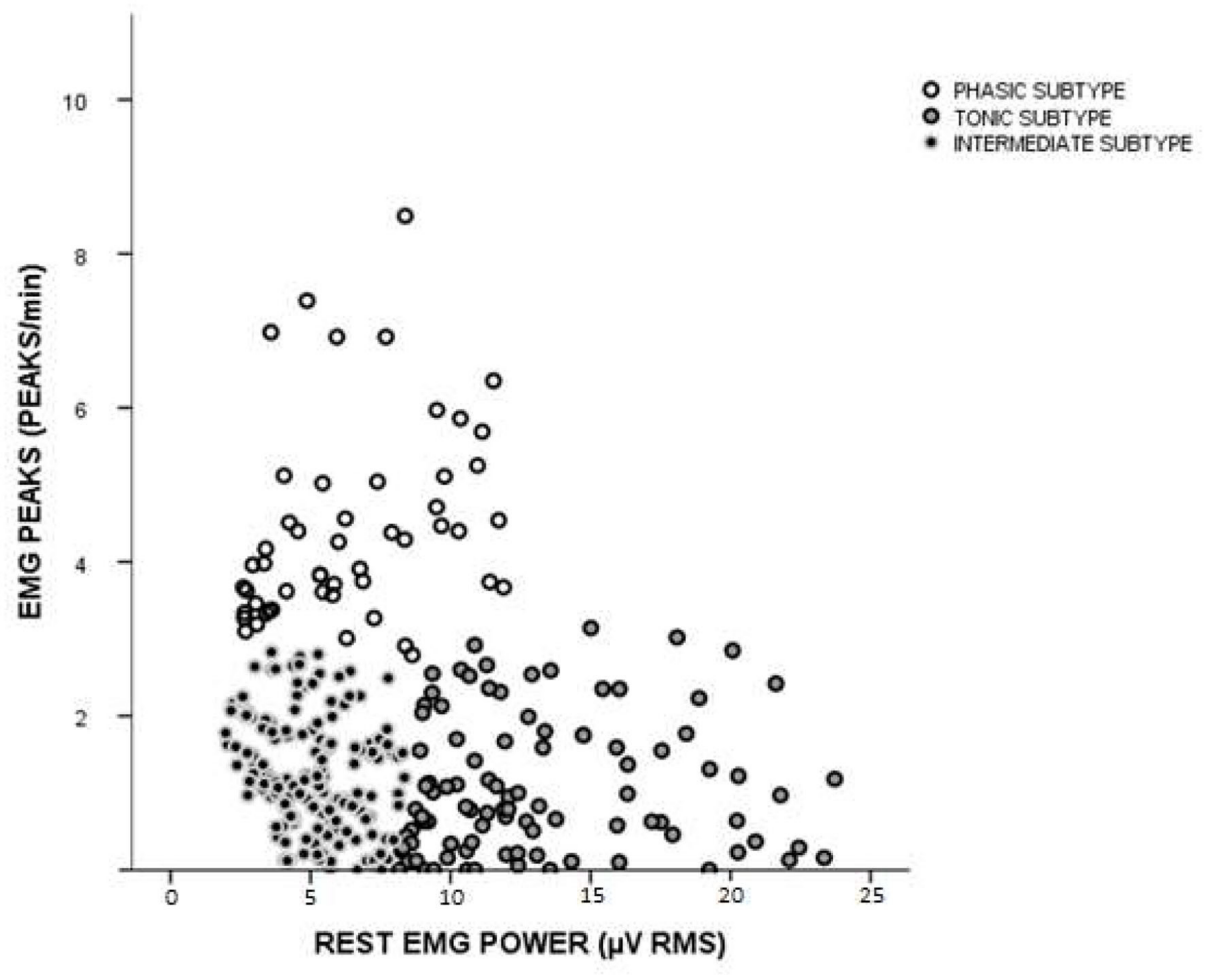
Cluster analysis of individuals with awake bruxism. Three clusters emerged from analysis: phasic (increased EMG peaks/min and less rest muscle tonus), tonic (increased rest muscle tonus and decreased EMG peaks/min), and an intermediary group.

Analyzing the EMG data of only the 308 participants with awake bruxism, as determined by clinical evaluation, revealed three clusters (Figure 3) representing three distinct subtypes of awake bruxism. The measure of cohesion and separation was 0.6 and considered good for cluster analysis. Individuals with the highest number of phasic contractions per minute and lowest EMG power were classified as the “phasic” subtype (51 participants—16.8%). Those with the highest rest EMG power and the smallest number of phasic contractions were classified as the “tonic” subtype (98 participants—32.3%). Those in whom both variables remained low were classified as the “intermediate” subtype (154 participants—50.8%).

**Figure 4:**
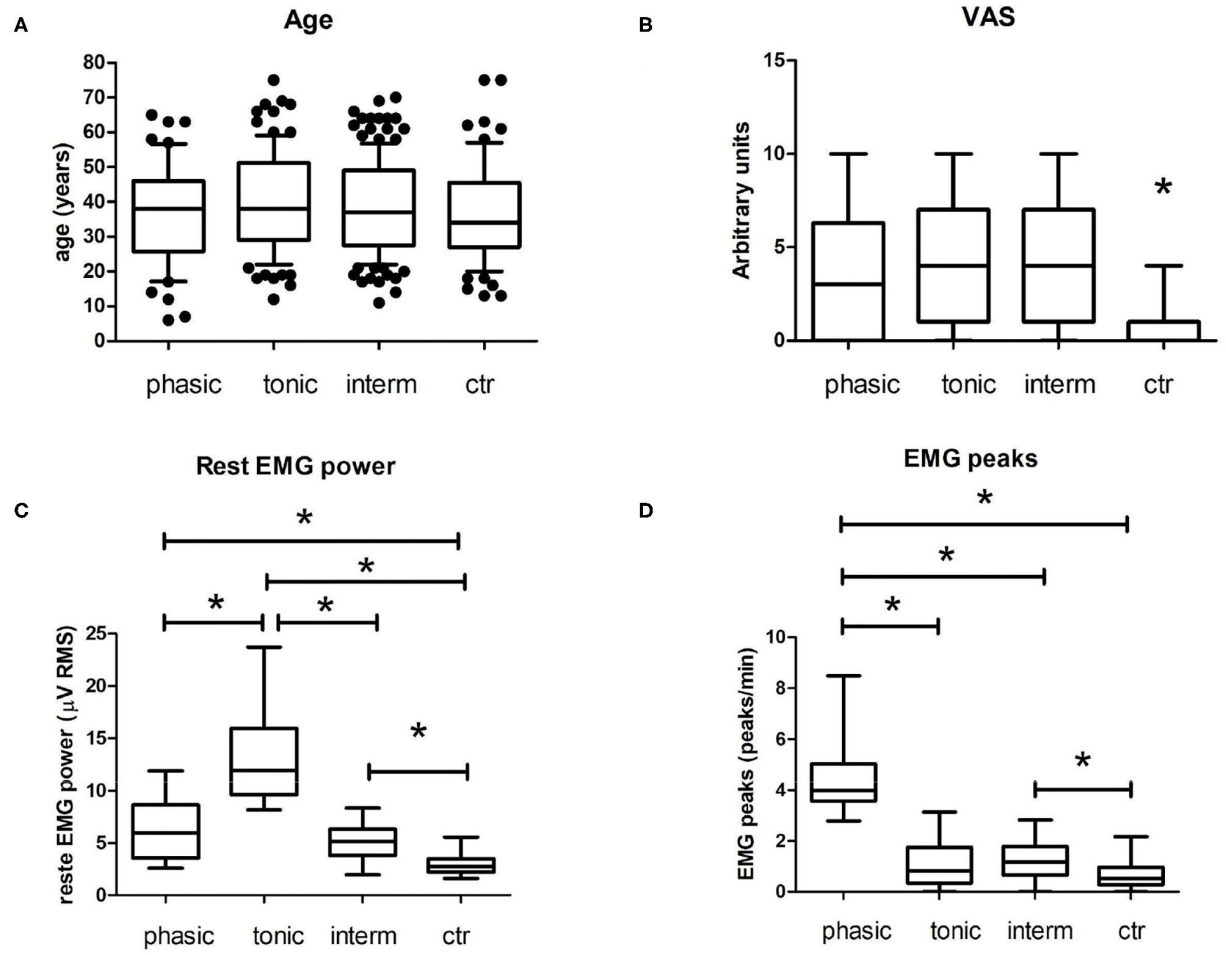
Main results of intergroup analyses. **(A)** Age of subjects included in each group. These boxplots represent median (horizontal line) and the 10th and 90th percentiles. Black dots represent points out of this range (outliers). There is no specific skewing for children or elderly in these groups. Also, there is no statistically significant difference between them (*p* > 0.05, One Way ANOVA with Tukey's Multiple Comparison *post-hoc* test). **(B)** Boxplot of self-reported pain in a Visual Analogical Scale (VAS, arbitrary units) from 0 (no pain) to 10 (intense pain). Ctr, control group. Ctr was different from all other groups. **(C)** Rest EMG Power. The tonic subgroup has a higher rest EMG power compared to other groups. **(D)** EMG peaks. The phasic subgroup has a higher peak/min range compared to other groups; *p < 0.05, Kruskal–Wallis with Dunn's Multiple Comparison *post-hoc* test; Data with normality checked with Kolmogorov–Smirnov test. The boxplots in **(B–D)** represent median (horizontal line) and minimum and maximum values.

Figure 4 and Table 1 show the descriptive statistics for all groups included in the study. With respect to age (Figure 4A), there is no statistically significant difference between groups. Also, no skewing was detected regarding child or teenage incidence between the groups. This can be seen in the boxplot of 10th to 90th percentiles. Pain intensity (Figure 4B), measured by the VAS, did not differ between awake bruxism subgroups (phasic, tonic, and intermediate); however, all three subgroups were significantly higher than the control group. The rest EMG power (Figure 4C) was significantly higher in the tonic subgroup compared to all other groups (including controls), but also the phasic and intermediary subgroups had significantly higher values than controls. With regard to the EMG peaks/min (Figure 4D), the phasic subgroup had significantly higher values than all groups, but also the intermediary subgroup was higher than controls.

Table 1 contains the participant characteristics. The 95% confidence interval (CI) unveils that all groups had similar distribution with regard to age, but also that we found a higher prevalence of bruxism in the female gender.

## Discussion

The main finding of the present study is that individuals included in the assessment of awake bruxism can be classified based on surface EMG characteristics into three different subtypes: phasic, tonic, and intermediate. However, if one looks at Figures 2, 3, it can be seen that some overlap exists between the intermediate subtype and control group. But the authors do not believe that EMG could be overidentifying patients. First, in the present work, EMG was not used for awake bruxism identification. Second, VAS pain data are statistically different between these two groups (4.1 ± 3.1 for intermediate awake bruxism and 0.48 ± 0.9 for controls, see also Figure 4B). We cannot rule out that some controls may be subclinical notifications of awake bruxism. When looking at the raw data, there was only one VAS score 4 in the control group. The upper limit of the 95% confidence interval (CI) of the mean in the control group is 0.68, quite below the apparently spurious score 4 occasionally found. With regard to pain, all three awake bruxism subtypes can be associated with some moderate pain; there is no difference between them, but all are higher than controls (see Figure 4B). One lesson that can be learned from this finding is that EMG alone is not (and will not) be the sole evaluation parameter of awake bruxism, as the clinical analysis is not (and will not be), either. Both approaches must be combined to gain a greater comprehension of what is most important: accurately evaluate the patient with regard to his/her physiology and possible awake bruxism assessment and predict the most suitable clinical approach for the best outcome possible, regardless if the patient has pain or not at the present time.

We believe that the side of sensor positioning can be aleatory. The criteria adopted here had just operational purposes. There is no description of laterality in awake bruxism. Also, people with unilateral masticatory muscle pain show no significant difference in muscle electrical activity of painful and non-painful muscles (Manfredini et al., 2013b). Furthermore, we compared right and left side EMG in 10 subjects of different subtypes, and did not find any difference (data not shown).

In a review of current methods for the assessment of bruxism, Pigozzi et al. (2019) point out the need for novel, more effective techniques. Current clinical methods require rigorous standardization and training and are still influenced by the subjective opinion of the examiner. According to Guillot et al. (2019), there is considerable disparity among health professionals regarding the assessment of bruxism, which results from different and often erroneous approaches to treatment. Inexperienced examiners, for example, may induce false pain when vigorously performing palpation on the muscle being evaluated.

Methods using portable devices (Yamaguchi et al., 2020), similar to those employed in the present study, have been gaining ground due to the increasing preference for evaluating individuals in their daily settings outside the clinic, which can alter the state of wakefulness and stress. Comparing EMG recordings under laboratory conditions to natural environment conditions, Prasad et al. (2019) found that differences in the amplitude of masseter muscle contractions were small and certainly not clinically relevant (0.94–1.00 to 0.82–1.00, respectively). Thus, the difference in evaluation setting does not exert influence over the degree of wakefulness, making surface EMG a reliable method in both situations (Prasad et al., 2019).

In the present study, no differences were found among the tonic, phasic, and intermediate subtypes regarding VAS scores. This finding contradicts our initial hypothesis that the tonic subgroup would have higher pain intensity values. However, individuals in the phasic and intermediate groups may have increased perceptions regarding pain symptoms, as individuals of different age groups and sexes were included in the sample. But a limitation of the present study was that the VAS score is subjective. We believe that further studies with a more specific methodology of pain evaluation (e.g., digital algometer) are necessary.

Future studies will be able to identify whether different psychomotor profiles and different brain circuits can influence the prevalence of phasic and tonic contractions in people with awake bruxism. In addition, understanding the different EMG patterns may assist in choosing specific treatments in cases where bruxism causes harmful consequences. According to Wetselaar et al. (2020), bruxism can be a risk factor with possible negative oral health outcomes, such as severe masticatory muscle pain or temporomandibular joint pain, extreme mechanical tooth wear, cracked teeth, and/or prosthodontic complications. It is also important to note that some of these oral changes are irreversible and progressive, such as tooth wear and cracked teeth. When tooth wear reaches advanced stages, it can cause pain, hypersensitivity, problems with function (e.g., chewing), and negative aesthetics (Maltarollo et al., 2020).

The international consensus in bruxism states that assessment of this condition may be performed by non-instrumental and instrumental approaches, such as physiological parameters acquired by EMG (Lobbezoo et al., 2018). There is also a recent review of the literature stating that masticatory EMG is important for the assessment of bruxism, which can be easily and precisely recorded during the daytime using a wearable EMG device (Yamaguchi et al., 2020). Here, we showed novel evidence that people included in the assessment of awake bruxism have different EMG patterns. The resultant EMG-based subtype classification may help establish new approaches for awake bruxism assessment, identifying effective treatment strategies, and improving outcome prognostication.

## Conclusions

The use of surface EMG enabled the identification of three distinct subtypes of awake bruxism (tonic, phasic, and intermediate), with no significant differences in VAS scores between them.

## Data Availability Statement

The raw data supporting the conclusions of this article will be made available by the authors, without undue reservation.

## Ethics Statement

The studies involving human participants were reviewed and approved by Comitê de ética em pesquisa com seres humanos—CEP UFPE. Written informed consent to participate in this study was provided by the participants' legal guardian/next of kin.

## Author Contributions

UM and VS: term, conceptualization, methodology, formal analysis, investigation, data curation, writing - original draft, writing - review and editing, and project administration. CS and TP: validation, investigation, and writing - review and editing. RX: term, conceptualization, methodology, validation, resources, writing- original draft, and writing - review and editing. MA: conceptualization, methodology, resources, writing - review and editing, and funding acquisition.

## Conflict of Interest

The authors declare that the research was conducted in the absence of any commercial or financial relationships that could be construed as a potential conflict of interest.
